# Artificial intelligence in tongue diagnosis: classification of tongue lesions and normal tongue images using deep convolutional neural network

**DOI:** 10.1186/s12880-024-01234-3

**Published:** 2024-03-08

**Authors:** Burcu Tiryaki, Kubra Torenek-Agirman, Ozkan Miloglu, Berfin Korkmaz, İbrahim Yucel Ozbek, Emin Argun Oral

**Affiliations:** 1 Department of Electrical Electronic Engineering, Faculty of Engineering, Ataturk University, Erzurum, Turkey; 2 Department of Oral Diagnosis and Dentomaxillofacial Radiology, Faculty of Dentistry, Ataturk University, Erzurum, Turkey; 3 Department of Electrical Electronic Engineering (High Performance Comp Applicat & Res Ctr), Ataturk University, Erzurum, Turkey; 4 Department of Oral, Dental and Maxillofacial Radiology, Faculty of Dentistry, Ataturk University, Erzurum, 25240 Turkey

**Keywords:** Deep convolutional neural network, Fusion based on majority voting, Tongue lesions

## Abstract

**Objective:**

This study aims to classify tongue lesion types using tongue images utilizing Deep Convolutional Neural Networks (DCNNs).

**Methods:**

A dataset consisting of five classes, four tongue lesion classes (coated, geographical, fissured tongue, and median rhomboid glossitis), and one healthy/normal tongue class, was constructed using tongue images of 623 patients who were admitted to our clinic. Classification performance was evaluated on VGG19, ResNet50, ResNet101, and GoogLeNet networks using fusion based majority voting (FBMV) approach for the first time in the literature.

**Results:**

In the binary classification problem (normal vs. tongue lesion), the highest classification accuracy performance of 93,53% was achieved utilizing ResNet101, and this rate was increased to 95,15% with the application of the FBMV approach. In the five-class classification problem of tongue lesion types, the VGG19 network yielded the best accuracy rate of 83.93%, and the fusion approach improved this rate to 88.76%.

**Conclusion:**

The obtained test results showed that tongue lesions could be identified with a high accuracy by applying DCNNs. Further improvement of these results has the potential for the use of the proposed method in clinic applications.

## Introduction

Tongue diagnosis is a noninvasive and convenient method for assessing human health, and its visual examination constitutes one of the main steps in oral diagnosis [1, 2]. Therefore, people in need of health care can expect a routine tongue examination during a health assessment [3]. Various studies in the literature evaluate tongue features such as its color, fur color, fur thickness, moisture, shape, teeth marks, holes, fissures, and stains to evaluate health status [1, 3]. There are many studies in the literature to assess different systemic diseases evaluating tongue features. Among them, prediabetes and/or diabetes [4–6], gastric cancer [7–9], esophageal cancer [10], and colorectal cancer [11] have been studied in evaluating tongue features.

The need for objective diagnostic methods has increased since clinical evaluation is subjective and depends on the physician’s experience. In recent years, the integration of artificial intelligence (AI) applications into the healthcare system has provided physicians with tools for objective evaluations. Gomes et al. [12] classified oral lesion images into 6 classes using clinically obtained images of basic lesions. They used ResNet50, Vgg16, InceptionV3, and Xception-based transfer learning models for classification. Islam et al. [13] used VGG19, DeIT, and MobileNet deep learning (DL) algorithms to classify oral lesions. Keser et al. [14] developed a DL approach to identify oral lichen planus lesions using photographic images and performed classification on all test images for both healthy and diseased mucosa images using Google Inception V3 architecture. Welikala et al. [15] used ResNet101 and Faster-RCNN DL models to detect malignant lesions and their classification.

In this study, lesion types of fissured tongue (FT), coated tongue (CT), geographic tongue (GT), and median rhomboid glossitis (MRG) along with healthy/normal tongue (NT) images are classified utilizing various DCNN with transfer learning. Also, majority voting is applied for the first time in the literature to improve the classification performance of tongue lesions. To evaluate the performance of the proposed classification approach, a new tongue lesion image dataset was constructed. All images were gathered from a specific medical center. Also, this dataset includes rare CT and MRG lesion types, and it has the potential to be used as a benchmark for this area.

## Methods and material

The Atatürk University Faculty of Dentistry’s Research Ethics Committee accepted the study, and all procedures were followed in accordance with the Declaration of Helsinki’s principles (Decision No. 04/2021) and informed consent was obtained from the patients for this study.

### Dataset

**Fig. 1:**
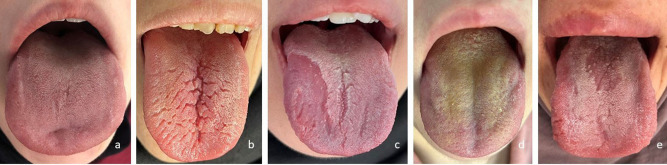
Examples of tongue lesions images: (**a**) normal/healthy tongue; (**b**) fissured tongue; (**c**) geographic tongue; (**d**) coated tongue; (**e**) median rhomboid glossitis

In the study, a new dataset was constructed and employed for the classification of tongue lesions. The dataset samples consist of images taken from patients who admitted to Faculty of Dentistry, Atatürk University for various dental problems. This dataset has 5 classes, of which 4 classes represent tongue lesions and 1 class represents NT images. They classes are briefly described below:

- NT; is pink in color, of medium thickness, without fissures, and has a slightly white and moist structure [16] as shown in Fig. 1a.

- FT (scrotal tongue, folded tongue, lingua plicata, tongue crack); is a common normal variant of the tongue surface. There are fissures of varying depths on the dorsal surface of the tongue, extending to the margin and limited to the anterior two-thirds [17] as shown in Fig. 1b.
- GT; is a benign, often asymptomatic, inflammatory condition of unknown cause that most commonly affects the dorsal aspect of the tongue. Especially in people who smoke, drink excessively, or have poor oral hygiene, shorter duration of lesions and more localized lesions may indicate malignancy [18]. A sample image is shown in Fig. 1c.
- CT (hairy tongue); is a benign condition caused by elongation of filiform papillae due to keratin deposition and is usually asymptomatic. It appears as a hairy covering on the dorsum of the tongue that protects the tip and lateral edges. Color depends on external factors such as diet smoking, and chromogenic bacteria, and varies from cream to brown and black, depending on internal factors such as fungi [19] as shown in Fig. 1d.
- MRG; is characterized by papillary atrophy located at the back of the tongue, typically in front of the circumvallate papillae. It appears as a well-circumscribed area of papillary atrophy in the midline of the tongue, in the shape of an ellipse or rhombus [20].. A sample image is shown in Fig. 1e.

Tongue image sample collection during the dataset creation process should meet some characteristics [21, 22]. Despite standardized tongue-imaging training, abnormal tongue images are nonetheless frequent in clinical tongue-imaging, both from operators and participants. These criteria are all considered during the construction of this new dataset with 623 tongue images. Their distribution over the classes is shown in Table 1. All dataset images were converted into joint photographic experts group (JPEG) format, and they have been resized based on the utilized network.

**Table 1:** Distribution of the number of data by classes

Classes	Coated	Fissured	Geographic	Median	Normal
Number of Data	84	142	175	67	155

In the labeling step of the constructed dataset, two oral diagnosis and dentomaxillofacial radiology experts, one with over 20 years of clinical experience, independently labeled all tongue images determined to be of good quality. Then, the two experts jointly labeled a small number of images with unmatching labels, and finally, for images where consensus could not be reached, a dermatologist was consulted.

### Classification networks and transfer learning

**Fig. 2:**
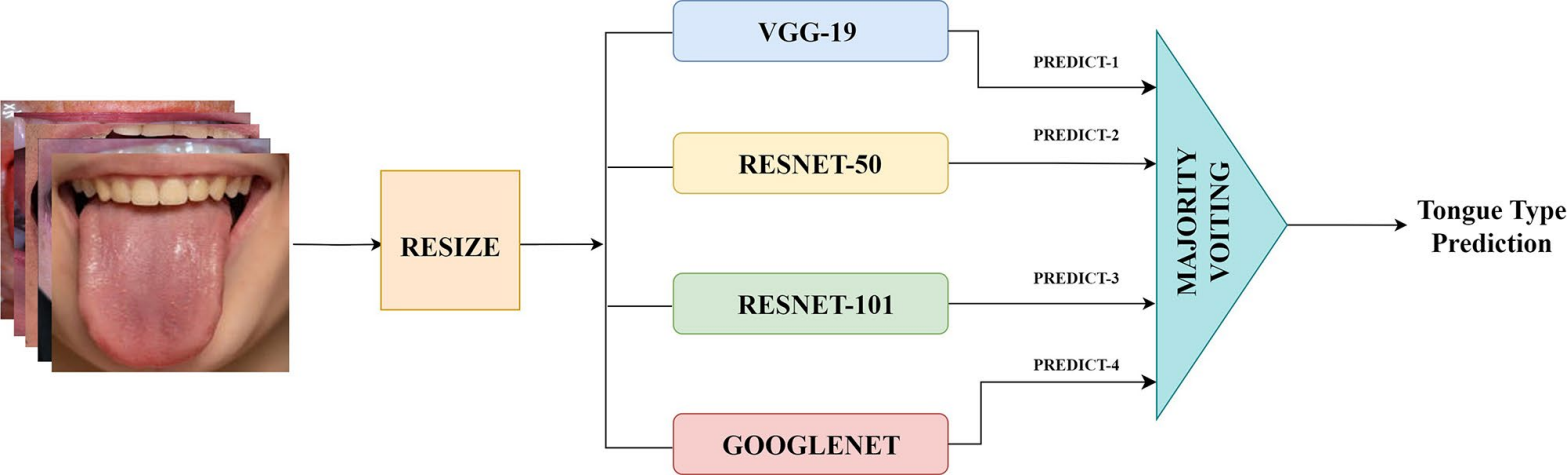
Block diagram of study

The block diagram of the study is shown in Fig. 2. It consists of three main blocks of resizing, classification, and majority voting to obtain the final tongue image classification. Four different DCNNs were utilized in the proposed study for tongue classification. Since these networks require a great amount of data during the training step, a transfer learning approach was utilized to tailor these networks for tongue classification with the available moderate amount of data. Transfer learning is using the knowledge, gained from one task, in others. This helps to tackle tasks using DL and machine learning algorithms [23].

DCNNs, employed in the study, are briefly described here.

#### VGG19

The architecture VGG19 consists of 3 fully connected layers, 16 convolution layers, 1 SoftMax layer, and 5 MaxPool layers. The number of filters in the convolution layers includes 64, 128, and 256 [24].

#### ResNet50

The ResNet50 architecture consists of 5 parts, and each part consists of a convolution block and an identity block containing 3 convolution blocks. It consists total of 50 neural network layers. In the ResNet50 architecture skip connections are used to feed output from one layer to the next [25].

#### ResNet101

The ResNet101 architecture consists of 33-layer blocks. Among these, 29 blocks directly use the output of the previous block, while the remaining 4 blocks utilize the output of the previous block in a convolutional layer with a filter size of 1 × 1 [26].

#### GoogLeNet

The GoogLeNet architecture includes a total of 22 layers. It consists of three different filters 1 × 1, 3 × 3, and 5 × 5 as described in [27].

These networks were pre-trained using a 1000-category ImageNet dataset containing more than 10 million images and designed for visual object recognition problems. During the transfer learning process, all layers of the networks except their last three layers have remained the same. In contrast, these last three were replaced with Fully Connected Layer, Softmax Layer, and Classification Layer. The output size of all networks was replaced with either 2 or 5 depending on the classification problem.

### Fusion of classification decisions using majority voting

In the fusion approach, the voting process is utilized to combine multiple classifier decisions to obtain a better classification performance. One of the common approach is the fusion based on majority voting (FBMV) that assigns a particular sample to its frequently observed class identity. That is, the mode value of decisions obtained from multiple networks is assigned as the label of a particular sample [28]. In majority voting based fusion step of the proposed study, a label is assigned for a particular test sample prediction if three or more networks predict the same class label for this sample. In case of equality as a result of the even number of employed classification networks, the class label for the sample is assigned randomly.

### Experimental setup

During the experimental studies, all images in the dataset were resized according to the network input layer requirement as the pre-processing step. Momentum Stochastic Gradient Descent (SGDM) was used as the optimization algorithm in each model. Additionally, to achieve high classification performance, MinibatchSize, Validation Frequency, InitialLearnRate, and Epoch hyperparameters were adjusted. These hyperparameter values, used for each model, are shown in Table 2. Also, 5-fold cross-validation was applied to avoid overfitting.

**Table 2:** Hyperparameters of models

	Hyperparameters				
MODEL	Mini-Batch Size	Optimizer	Max Epoch	Initial Learn Rate	Val Freq
VGG19	5	SGDM	200	3e-4	50
RESNET50	5	SGDM	100	3e-4	50
RESNET101	5	SGDM	50	3e-4	100
GOOGLENET	5	SGDM	100	3e-4	100

SGDM: Momentum Stochastic Gradient Descent

During all experiments, studies were implemented on a 64-bit Ubuntu 18.04 system with 128GB RAM and NVIDIA GeForce RTX 2080 TITAN graphics processing unit.

### Evaluations metrics

The Confusion matrix is used to measure the quality of classification performance, and it was used in the current study to evaluate the performance of four DCNNs. The major components of a binary confusion matrix are given in Table 3 [29]. This matrix defines the counts of true positive (TP), false positive (FP), false negative (FN), and true negative (TN) tests. Based on these test results, Accuracy, Sensitivity, Specificity, Precision, Recall and F1 Score test metrics are obtained as given as follows.

**Table 3:** A confusion matrix for binary classification

		Predict	
		**Positive**	**Negative**
True	**Positive**	TP	FN
	**Negative**	FP	TN

TP: True positive; TN: True negative; FP: False positive; NF: False negative

1 $$ Accuracy=\frac{TP+TN}{TP+FP+FN+TN}$$

2 $$ Sensitivity=\frac{TP}{FN+TP}$$

3 $$ Specificity=\frac{TN}{TN+FP}$$

4 $$ Precision=\frac{TP}{TP+FP}$$

5 $$ Recall=\frac{TP}{TP+TN}$$

6 $$ {F}_{1}=2*\frac{Precision*Recall}{Precision+Recall}$$

## Results

In the first step of the study, the tongue lesion dataset was divided into two classes of “normal/healthy” and “lesion”, both with 155 samples to form a balanced data distribution. The 2-class classification process was performed on four DCNNs. The obtained accuracy metrics of all models for each fold are shown in Table 4. As can be seen from this table, the highest success rate was achieved for ResNet101 with 93.53% accuracy while that of the lowest was 89,83% with GoogLeNet. As a result of the FBMV, on the other hand, the accuracy improves to 95.15%.

**Table 4:** Accuracy values of models for binary classification in each fold

MODELS/FOLDS	FOLD1	FOLD2	FOLD3	FOLD4	FOLD5	AVERAGE (%)
VGG19	91,94	91,94	86,89	93,55	88,71	90,61
RESNET50	85,48	93,55	90,16	90,32	91,94	90,29
RESNET101	93,55	95,16	93,44	91,94	93,55	**93,53**
GOOGLENET	88,7	95,1	88,52	87,1	88,71	89,83
FUSION						**95,15**

In the second step of the study, classification was performed on the 5-class dataset. The obtained accuracy results of each network are shown in Table 5, given for all folds. It is clear from this table, that the highest success rate was obtained in the VGG19 model with 83.93%. Applying FBMV, it improves to 88.76%, as expected.

**Table 5:** Accuracy values of models for multi class classification in each fold

MODELS/FOLDS	FOLD1	FOLD2	FOLD3	FOLD4	FOLD5	AVERAGE (%)
VGG19	81.45	80.15	82.92	88.88	86.62	**83.93**
RESNET50	79.03	79.36	79.67	83.33	83.06	80.89
RESNET101	81.45	83.33	79.67	84.12	81.45	82
GOOGLENET	77.41	84.92	81.3	83.33	82.25	81.84
FUSION						**88.76**

**Fig. 3:**
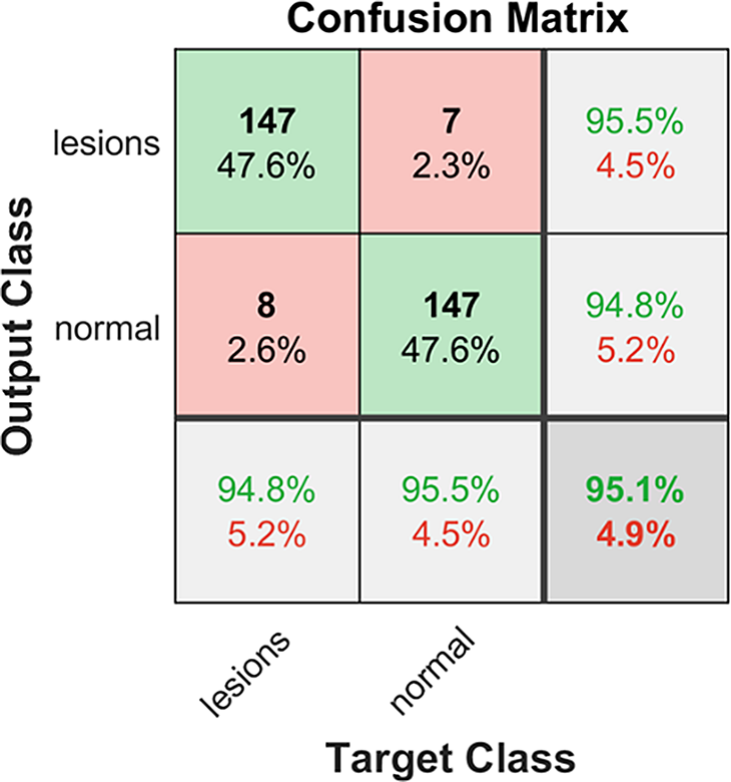
Confusion matrix of fusion in binary classification

**Fig. 4:**
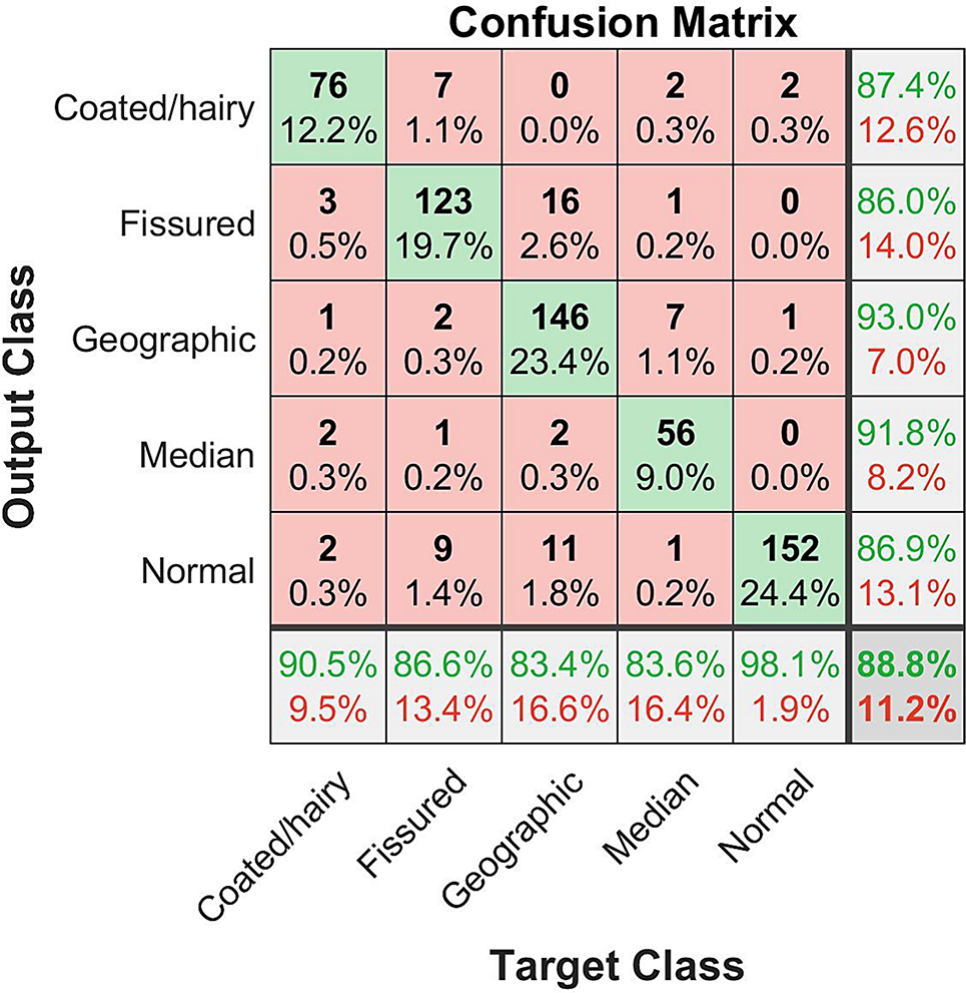
Confusion matrix of fusion in multi class classification

The confusion matrix of both binary-class and multi-class classification test results are shown in Figs. 3 and 4, respectively. True labels versus predicted labels are shown in both confusion matrices. The sum of the entries in each row represents the count of the data for this specific class. The numbers on the diagonal, on the other hand, shown in green represent the number of data correctly estimated while non-diagonal entries indicate the number of incorrectly estimated data. For example, there is a total of 84 data in the CT class. While 76 of these are classified correctly, incorrectly estimated data counts were 3, 1, 2, and 2 for FT, GT, MRG, and NT classes, respectively. The most errors were obtained in the GT class with 29 data, while the least errors were obtained in the NT class with 3 data, as shown in Fig. 4.

While evaluating the accuracy performance of the DCNNs, accuracy, sensitivity, specificity, and F1 score test metrics were evaluated as the data distribution among the classes is unbalanced in the dataset. In addition to this, average accuracy values were obtained, and all these test results are shown in Table 6. Among all networks, the highest accuracy rate of 90,64% was obtained in the GT class using ResNet50. As a result of the fusion, the accuracy rate improves to 92,99%. Sensitivity value was also evaluated for each class in all networks. ResNet50 produced 96.13%. the highest sensitivity rate in the NT class while it was improved to 98.06% as a result of fusion. While the highest specificity value of 98.38% was obtained in the MRG among all five classes with the use of VGG19 and ResNet101 networks, the application of the fusion increases this score to 99.1%. Finally, the highest F1 score of was obtained as 88.73% and 92.12% with ResNet50 and application of the fusion process, respectively. These obtained test results show the effectiveness of the proposed method in the tongue lesion classification problem.

**Table 6:** Accuracy, Sensitivity, Specificity and F1 Score values in each model of 5 classes

MODELS	CLASSES	ACCURACY	SENSITIVITY (%)	SPECIFICITY (%)	F1 SCORE (%)
VGG19	Coated/Hairy	83,53	84.52	97.40	84.02
	Fissured	82,10	83.80	94.59	82.93
	Geographic	85,03	81.14	94.42	83.04
	Median	84,48	79.10	98.38	82.17
	Normal	84,15	89.03	94.44	86.52
RESNET-50	Coated/Hairy	76,54	73.81	96.47	75.15
	Fissured	80,85	72.00	97.10	80.25
	Geographic	90,64	86.90	97.10	88.73
	Median	80,30	79.10	97.66	79.70
	Normal	76,02	96.13	89.96	84.90
RESNET-101	Coated/Hairy	78,89	84.52	94.47	81.61
	Fissured	81,12	81.69	94.39	81.40
	Geographic	86,36	76.00	95.31	80.85
	Median	84,21	71.64	98.38	77.42
	Normal	79,89	92.26	92.31	85.63
GOOGLENET	Coated/Hairy	81,18	82.14	97.03	81.66
	Fissured	78,47	79.58	93.56	79.02
	Geographic	84,47	77.71	94.42	80.95
	Median	79,17	85.07	97.30	82.01
	Normal	83,85	87.10	94.44	85.44
FUSION	Coated/Hairy	87,36	90.48	97.96	88.89
	Fissured	86,01	86.62	95.84	86.32
	Geographic	92,99	83.43	97.54	87.95
	Median	91,80	83.58	99.10	87.50
	Normal	86,86	98.06	95.09	92.12

## Discussion

Blending old information with new technological developments makes artificial intelligence technology more functional for physicians. In recent years, DL methods have been widely studied in various applications including tongue diagnosis. They provide objective and quantitative evaluation and facilitate physicians in the differential diagnosis of tongue lesions. In this regard, DL based tongue segmentation, tongue-type classification, and tongue related disease identification have been studied in the literature [30]. Among these, FT [20, 31], tooth-marked tongue [32, 33], tongue prickles [34], recognition performance, tongue image standardizing [5, 21], classification of tongue features such as color, movement, shape [1, 3, 8, 35], and tongue-coating systemic-disease relationship [4, 5, 7, 8, 36, 37] studies suggest promising results.

The proposed study has several unique aspects that distinguish it from existing literature. It employs state-of-the-art DL methods, is a multi-class study, and suggests high-accuracy performance. Additionally, it involves the classification of both CT and MRG and uses a dataset that is robust against inter-sample variation.

In terms of applying modern DL methods, Yang et al. [38] have developed intelligent tongue diagnosis systems that employ DL methods to quickly and accurately identify tongue pathological features. They utilized YOLOv5s6, U-Net, and MobileNetV3 models for tongue recognition, tongue region segmentation, and tongue feature classification, respectively. Classification accuracy rates for teeth marks, stains and fissures were obtained as 93.33%, 89.60% and 97.67%, respectively. Heo et al. [39], DL was used to identify tongue cancer patients extracting 5576 tongue images obtained from 12,400 endoscopic images. DenseNet169, the best model, yielded an AUROC value of 0.895 and an AUPRC value of 0.918. In another study to classify and detect oral potential malignant disorders (OPDM) that turn into oral cancer, AUC was obtained as 95% in DenseNet121 and ResNet50 models used for two-class classification using 300 OPDM and 300 normal oral mucosa images [40]. The detection performance of 74.34% AUC was achieved with R-CNN. In this regard, the proposed study was also performed through the use of a similar size dataset with 623 clinical images but it was implemented as a multi-class classification problem and it yielded an accuracy rate over 88%. Also, in two classes of normal tongue and other classification problems, 95% accuracy performance was obtained.

Hu et al. [30], in their retrospective study, developed a new framework, TongueNet, that performs better compared to InceptionV3 and ResNet18 in terms of the accuracy rate of detecting 721 FTs. In another retrospective study, Yan et al. [31] reported tongue crack extraction and recognition based on Segmentation-Based Deep Learning (SBDL) utilizing Mask R-CNN, DeeplabV3+, U-Net, UNet++, and SegAN algorithms on a tongue image dataset with 176 cracked-tongue samples and 140 crack-free samples. They stated that SBDL is effective in recognizing tongue fissures, and it solves the problem of removing incorrect tongue fissures that may arise from a few data sets. They also claimed that this strategy produces optimistic results for tongue crack removal and recognition. A similar problem of detecting FT but in a 5-class dataset was also performed in the current study. Four different networks, namely VGG19, ResNet50, ResNet101 and GoogLeNet, were employed to compensate for a network being delicate to a specific metric as a result of conducting experiments on a moderate-size dataset. The highest accuracy rate of 86.01% and sensitivity ratio of 86.62% using the fusion approach and the highest specificity ratio of 97.1% using ResNet50 were obtained.

Although there are studies in the literature that evaluated the color and thickness of tongue coating [11, 41], no specific study, to the best of our knowledge, was performed for CT the classification. The only study by Wang et al. [34] developed an oily tongue coating recognition approach using convolutional neural networks, and they obtained an accuracy rate of 88.8% on a tongue image dataset with 1486 samples. In the proposed study, the classification of 84 CT images was performed. The highest accuracy rate of 87.36% and the highest sensitivity ratio of 90.48% and the highest specificity rate of 97.96% using fusion were obtained.

Zhang et al. [42] reported that GT was significantly associated with FT, burning mouth syndrome, oral lichen planus, and gastrointestinal disorders, but not with systemic diseases such as recurrent aphthous ulcers or cardiovascular diseases. Shamim et al. [43] evaluated the tongue lesion classification performance of five classes, namely FT, GT, HT and two other rare precancerous tongue lesions, utilizing six DCNNs models applying transfer learning. Compared to this study, the proposed work not only standardizes the dataset but also employs FBMV in tongue lesion classification problem for the first time in the literature. A total of 175 GT samples were included in the study, and the highest sensitivity rate of %86.90 using ResNet50 and the highest accuracy and specificity values of 92.99% and 97.54% using fusion were obtained respectively.

MRG evaluation based on ML has not been studied in the literature. The current study, on the other hand, contains 67 MRG samples in the freshly proposed dataset, and the highest accuracy rate of 91.8% and highest specificity rate of 99.10% were obtained using fusion while GoogLeNet yielded the highest sensitivity ratio of 85.07%.

Finally, the classification of 155 NT images, included in the proposed study, resulted in the highest accuracy rate of 86.86%, sensitivity rate of 98.06%, and specificity rate of 95.09% all employing fusion.

In the proposed study, a five-class dataset, consisting of four different tongue lesions and normal/healthy tongue images, was created. This dataset includes rare CT and MRG lesion samples, and it has the potential to be used as a benchmark in this area as it is constructed using images of a specific medical center with less imaging variability. In the study, a new dataset was classified utilizing four DL approaches. Since this moderate-size dataset is unbalanced among the classes, a transfer learning approach was employed to compensate for this problem. Also, each DL model was processed through 5-fold cross-validation to assess the accuracy and generalizability of the predictive models as well as avoid from risk of overfitting. Despite all these limitations, the proposed approach shows a good performance in the tongue lesions classification problem.

## Conclusion

FBMV was used to perform a 5-class tongue lesion classification problem for the first time in the literature. The obtained classification accuracy performance was over 95% in the 2-class problem and 88% in the 5-class problem. In future, we plan to improve the classification performance by expanding the dataset samples as well as compensate for its unbalanced distribution. Also, it is planned to include new lesion classes in the dataset to increase the effectiveness of the current framework. By that, the proposed work can help medical professionals in clinical settings to diagnose and screen for tongue lesions.

## Data Availability

The datasets used and/or analyzed during the current study are available from the corresponding author on reasonable request.
